# Potential Prebiotic Effects of *Artemisia capillaris*-Derived Transglycosylated Product

**DOI:** 10.3390/foods13203267

**Published:** 2024-10-14

**Authors:** Heewon Moon, Keunsoo Kang, Misook Kim

**Affiliations:** 1 Department of Food Science and Nutrition, Dankook University, Cheonan 31116, Republic of Korea; hw1356@naver.com; 2 Department of Microbiology, Dankook University, Cheonan 31116, Republic of Korea; kangk1204@gmail.com

**Keywords:** prebiotic, gut microbiota, in vitro fermentation, transglycosylated product, *Lactobacillus*, *Salmonella*

## Abstract

This study investigated the impact of a transglycosylated product (ACOD) catalyzed by *Leuconostoc mesenteroides* MKSR dextransucrase using sucrose as a glucosyl donor and both maltose and *Artemisia capillaris* as acceptors on gut microbiota through fecal fermentation. ACOD promoted the growth of probiotics such as *Lactiplantibacillus plantarum*, *Lacticaseibacillus casei*, *Lacticaseibacillus rhamnosus* GG, and *Leuconostoc mesenteroides* MKSR, while inhibiting the growth of pathogenic bacteria such as *Escherichia coli*, *E. coli* O157:H7, *Enterococcus faecalis*, *Listeria monocytogenes*, *Staphylococcus aureus*, *Shigella flexneri*, *Streptococcus mutans*, *Pseudomonas aeruginosa*, and *Bacillus cereus* during independent cultivation. Fecal fermentation for 24 h revealed that ACOD significantly increased the production of short-chain fatty acids (SCFAs) compared to the blank and fructoooligosaccharide (FOS) groups. Specifically, ACOD led to a 4.5-fold increase in acetic acid production compared to FOSs and a 3.3-fold increase in propionic acid production. Both the ACOD and FOS groups exhibited higher levels of butyric acid than the blank. Notably, ACOD significantly modulated the composition of the gut microbiota by increasing the relative abundances of *Lactobacillus* and decreasing *Escherichia/Shigella* and *Salmonella.* In contrast, FOSs remarkably promoted the growth of *Salmonella*. These findings suggest that ACOD is a potential candidate for prebiotics that improve the intestinal environment by being actively used by beneficial bacteria.

## 1. Introduction

The human large intestine harbors a complex and metabolically active gut microbiota, which is composed of anaerobic bacteria classified mainly as Firmicutes, Bacteroidetes, and Proteobacteria. The gut microbiota produces profitable metabolites through the degradation and use of dietary substrates, such as short-chain fatty acids (SCFAs), which are beneficial for host physiology and energy homeostasis [1]. However, the delicate equilibrium of the human gut microbiota is susceptible to modification induced by dietary factors. Dietary interventions are considered as one of the crucial modulators of the human gut microbiota, influencing its composition. The intricate interplay between diet and the gut microbiota has been linked to various physiological and pathological conditions. While the impact of prebiotic dietary supplements as well as probiotic on the gut microbiota is well-established, there is growing interest in exploring the potential of various prebiotic materials [2,3,4,5,6,7].

Prebiotics are typically defined as “selectively fermented, nondigestible food ingredients or substances that specifically support the growth and/or activity of health-promoting bacteria that colonize the gastrointestinal tract” [8]. Furthermore, this definition of prebiotics indicates that although all dietary carbohydrates are not prebiotics, they must satisfy the following criteria: (1) resistance to gastric acidity (low pH), hydrolysis by mammalian enzymes, and gastrointestinal absorption; (2) fermentation by intestinal microflora; and (3) selective stimulation of the growth and/or activity of intestinal bacteria that contribute to health and wellbeing [9]. Nondigestible carbohydrates (NDCs), including dietary fibers and several oligosaccharides (such as fructans, lactulose, galactooligosaccharides, fructooligosaccharides (FOSs), isomaltooligosaccharide (IMOs), and human milk oligosaccharides), have been reported to exert a variety of prebiotic effects [10].

The prebiotic concept has still been limited to selected nondigestible carbohydrates. However, phytochemicals, such as phenolic compounds, can exert a prebiotic effect by selectively stimulating beneficial bacteria while reducing the occurrence of disease [11]. Phenolic compounds are a varied class of secondary plant metabolites present [12]. They are poorly absorbed during digestion due to their chemical composition and later reach the colon, where they influence the resident microbiota. More precisely, phenolic compounds have the capacity to stimulate several crucial bacterial species such as *Akkermansia muciniphila, Bacteroides thetaiotaomicrom, Faecalibacterium prausnitzii*, *Bifidobacteria* spp., and *Lactobacillus* spp. [13].

*Artemisia capillaris* is found mainly in temperate regions of Asia, Europe, North America, and Africa. It has been reported to exhibit antibacterial, antidementia, antidiabetic, and antiobesity effects in several studies due to its polyphenol-rich compounds [14,15,16]. However, one of the main disadvantages of these compounds is their usually poor bioavailability when administered to humans [17].

Transglycosylation, catalyzed by enzymes from various bacteria, has been used to improve physicochemical properties. This includes enhancing the solubility of water and the oxidative stability of various compounds [18]. In a previous study [19], the optimal conditions for *Leu. mesenteroides* MKSR dextransucrase-catalyzed transglycosylation were established to synthesize *A. capillaris*-derived oligosaccharide (ACOD) in the presence of sucrose as a glucosyl donor and maltose and water extracted *A. capillaris* (AC) as glucosyl acceptors. While previous research has demonstrated the synthesis of ACOD and its physiological characteristics, this study represents the first investigation into the prebiotic potential of ACOD and its effects on the gut microbiota. In addition, this study compares the impact of ACOD with a transglycosylation product of sucrose and maltose catalyzed by MKSR dextransucrase (OD) and the well-known prebiotic, fructooligosaccharides (FOSs), to evaluate ACOD’s ability to modulate gut microbiota composition through in vitro human fecal fermentation. The results provide novel insights into the potential application of ACOD as a prebiotic, emphasizing its unique influence on both pathogenic and beneficial bacteria. The gut microbiota plays a crucial role in host physiology. It is well known that an intimate symbiotic relationship exists between the gut microbiome and the host and that this association is complex and multidimensional, as it affects the gut–lung, gut–brain gut–skin, gut–muscle, and gut–adipose tissue axes, among others [20]. Therefore, the present study aimed to investigate the effects of ACOD on the growth of pathogenic and probiotic bacteria and on gut microbiota composition through in vitro human fecal fermentation.

## 2. Materials and Methods

### 2.1. Materials

A transglycosylated product was produced by *Leu. mesenteroides* MKSR dextransucrase (0.05 U) in the mixture of 3.59% *A. capillaris*, 182.54 mM sucrose, and 11.30 mM maltose in 20 mM sodium acetate buffer (pH 5.2) [19]. Enzymes such as α-amylase, pepsin, pancreatin, and bile salts were purchased from Sigma Aldrich (Saint Louis, MO, USA). *Escherichia coli* KCTC 1682, *Pseudomonas aeruginosa* KCTC 1750, *Shigella flexneri* KCTC 22192, *Staphylococcus aureus* KCTC 3881, *Enterococcus faecalis* KCTC 2011, *Listeria monocytogenes* KCTC 3710, *Streptococcus mutans* KCTC 3065, *Bacillus cereus* KCTC 1012, *Lacticaseibacillus paracasei* KCTC 3165, *Lactiplantibacillus plantarum* KCTC 3103, and *Lacticaseibacillus rhamnosus GG* KCTC 3237 were distributed from KCTC (Korean Collection for Type Cultures, Jeongeup, Korea), and *Escherichia coli* O:157 KCCM 40406 was obtained from KCCM (Korea Culture Center of Microorganisms, Seoul, Korea).

### 2.2. Simulated Saliva–Gastrointestinal Digestion

The digestion of ACOD was simulated in vitro for saliva–gastrointestinal digestion using a modified method described by Ding et al. [21] and Hwang and Kim [22]. Simulated salivary juice (1.0 L) consisted of Na_2_HPO_4_ (2.38 g), KH_2_PO_4_ (0.19 g), NaCl (8 g), and α-amylase (210.90 mg), with the pH adjusted to 6.8 using 0.1 M HCl. Initially, the sample solution was mixed with the simulated salivary juice (1:1, *v*/*v*) and then was reacted in a water bath shaker at 37 °C for 5 min. Subsequently, the simulated gastric juice, containing pepsin (0.32 g/100 mL) and NaCl (0.03 M), was added to the mixture (1:1, *v*/*v*), and the pH of the mixture was immediately adjusted to pH 2.0 using 0.1 M HCl. The mixture was also reacted in a water bath shaker at 37 °C for 2 h. Finally, the simulated small intestine’s juices, comprising pancreatin (0.15 g/100 mL) and bile salts (0.9 g/100 mL) in 0.1 M NaHCO_3_ adjusted to pH 6.8, was added to the mixture at a ratio of 3:10 (*v*/*v*). The solution was incubated at 37 °C for 2 h.

### 2.3. Determination of Total Carbohydrate, Phenolic, and Flavonoid Content

The total carbohydrate content was analyzed using the phenol-sulfuric acid method [23]. Subsequently, 1 mL of samples and 1 mL of a 5% (*w*/*v*) aqueous solution of phenol was prepared in a test tube. Next, 5 mL of concentrated sulfuric acid was added to the test tube containing the mixture, followed by placing the test tube at 30 °C for 30 min. The spectrophotometer measured light absorption at 470 nm, and the carbohydrate concentration was determined from a standard curve using maltose.

The determination of total phenolic compounds was performed according to the method by Chen et al. [24]. The supernatant (100 μL) from each digestion phase was mixed with 400 μL of distilled water and 100 μL of Folin–Ciocalten reagent. After 6 min, 1 mL of 7% (*w/v*) Na_2_CO_3_ and 0.8 mL of distilled water were added. The mixture was then kept at room temperature for 90 min, and the absorbance was recorded at 760 nm using a spectrophotometer. Total phenolic compounds were expressed as mg (milligrams) of gallic acid equivalents per milliliter (mL).

For total flavonoid quantification, the sample (1 mL) was mixed with 150 μL of NaNO_2_ and allowed to stand for 6 min at room temperature [24]. Next, 300 μL of AlCl_3_·6H_2_O was added and again incubated for 6 min at room temperature. Finally, 1 mL of 1 M NaOH and 1.05 mL of distilled water was added. The solution was mixed, and the result was read at 510 nm immediately. The total flavonoid content was calculated from a standard curve of quercetin (ug/mL) equivalent.

### 2.4. Growth Inhibition and Promotion of Single Bacterial Strains

The antibacterial inhibition activity assay was performed in a 96-well plate, following a modified method of Vijayakumar and Muriana [25]. Nine different pathogenic bacteria, namely, *E. coli*, *E. coli* O157, *Ent. faecalis*, *Lis. monocytogenes*, *Stap. aureus*, *Shi. flexneri*, *Strp. mutans*, *P. aeruginosa*, and *B. cereus,* were cultured in tryptic soy broth at 37 °C. Four probiotic bacteria, namely, *Lcb. rhamnosus*, *Lcb. paracasei*, *Lpb. plantarum*, and *Leu. mesenteroides* MKSR, were cultured in MRS broth at 30 °C. The sample (ACOD, AC, or OD) was then mixed with an equal volume of tryptic soy broth or MRS, separately, and each bacterium was inoculated at a final concentration of 6–7 log CFU/mL. After incubation for 24 h at the specified temperatures, the absorbance was measured at 600 nm.

### 2.5. In Vitro Fermentation Using Human Fecal Inoculum

Fecal fermentation of ACOD was performed using the reported method by Hajar–Azhari et al. [26] with modifications. Four volunteers with a healthy diet (two men and two females, 23–25 years old), who did not have digestive diseases or antibiotic treatment in the past 6 months, were the donors of fresh fecal samples. The fecal samples were collected and diluted with 0.1 M phosphate-buffered saline to obtain 10% fecal slurry (*w*/*v*). After centrifugation, 1.5 mL of fecal slurry was mixed with 8.5 mL of basal nutrient medium (yeast extract (2.0 g/L), peptone (1.0 g/L), NaHCO_3_ (2.0 g/L), bile salts (0.5 g/L), NaCl (0.1 g/L), K_2_HPO_4_ (0.04 g/L), KH_2_PO_4_ (0.01 g/L), CaCl_2_·2H_2_O (0.01 g/L), MgSO_4_·7H_2_O (0.01 g/L), hemin (0.02 g/L), L-cysteine (0.5 g/L), resazurin solution (1.0 mL/L, 1%, *w*/*v*), Tween 80 (2.0 mL/L), and vitamin K (10.0 μL/L)) with 5 mL of ACOD or fructooligosaccharides (FOS group) or without carbohydrates (blank group). The fecal fermentation was performed for 24 h at 37 °C in a thermostatic shaker. Anaerobic conditions were maintained throughout fecal fermentation.

Genomic DNA was extracted from the sample using the Dneasy PowerSoil Pro Kits (QIAGEN, San Diego, CA, USA), ensuring the isolation of high-quality DNA suitable for downstream applications. The DNA concentration was then measured using both the Nanodrop spectrophotometer and Qubit fluorometer to ensure accurate quantification. To target the hypervariable V3–V4 region of the 16S rRNA gene, specific fusion primers (Illumina_16S_341F: 5′-TCGTCGGCAGCGTCAGATGTGTATAAGAGACAGCCTACGGGNGGCWGCAG and Illumina_16S_805R: 5′-GTCTCGTGGGCTCGGAGATGTGTATAAGAGACAGGACTACHVGGGTATCTAATCC) were used for PCR amplification. The PCR products were then combined with a multiplexing index and Illumina sequencing adapters to prepare a sequencing library according to the Illumina 16S Metagenomic Sequencing Library Preparation protocol. This process resulted in a final library product that included both the Illumina adaptors and indices and was generally less than 630 bp in size. Finally, paired-end sequencing (2 × 300 bp) was performed using the MiSeq platform (Illumina Inc., San Diego, CA, USA) to generate high-resolution microbial community data [27,28].

### 2.6. Determination of Short-Chain Fatty Acids during In Vitro Fermentation

The LC–MS/MS analysis utilized an integrated system, comprising Agilent 1260 Infinity Binary LC and Agilent 6420 Triple Quadrupole LC/MS (Santa Clara, CA, USA). The reaction mixture of GT-labeled SCFA, with a volume of 5 µL, was injected into an Agilent Zorbax HILIC Plus column (4.6 × 100 mm, 3.5 µm). The mobile phase consisted of two solvents: Solvent A, which contained water with 20 mM ammonium acetate and 20 mM acetic acid, and Solvent B, which was 100% acetonitrile. The separation of GT-labeled SCFAs occurred on the analytical column at a flow rate of 500 µL/min. The LC gradient method was established as follows: at t = 0 min, 70% B; at 2 min, 70% B; at 12 min, 60% B; at 12.5 min, 70% B; and at 19 min, 70% B. The mass spectrometer operated in positive ion mode, with an electrospray ionization spray voltage of 4 kV and a capillary temperature of 300 °C [29].

### 2.7. Statistical Analysis

Each experiment was carried out in triplicate, and the results were expressed as mean ± standard deviation. One-way analysis of variance was performed using XLSTAT software version 2012 (Addinsoft Inc., Paris, France). The significance analysis was performed using the Tukey test, and *p* < 0.05 was considered statistically significant.

## 3. Results and Discussion

### 3.1. Impact of Saliva–Gastrointestinal Digestion on ACOD

Prebiotics are nondigestible compounds that resist upper gastrointestinal tract digestion, are fermented by beneficial bacteria in the colon, and selectively promote the growth and activity of these beneficial microorganisms, ultimately contributing to a healthier gut environment and overall host health [30]. An *A. capillaris*-derived transglycosylated product is a mixture composed of isomaltooligosaccharides, dextran, and AC-derived compounds that is produced using sucrose as the donor substrate and maltose and AC extract as the acceptor substrates [31]. There was no difference in the total carbohydrate content of ACOD before and after digestion (Table 1). Most plant-derived non-starch polysaccharides resist digestion by human saliva but undergo hydrolysis in the low pH environment of gastric digestion. Additionally, the presence of free monosaccharides can be the result of the disturbance of aggregates caused by gastric acid. Enzymes released by the intestinal microbiota can decompose most indigestible polysaccharides into reducing sugars, serving as sources of carbon for the intestinal microbiota [32]. Our findings suggested that the release of polyphenols and flavonoids was lower before saliva–gastrointestinal digestion in vitro. Lima et al. [33] also reported that the total phenolic content and total flavonoid content of *Artemisia gorgonum* decreased after in vitro gastrointestinal digestion. The slight reduction in phenolics observed during the gastric phase may be attributed to the potential formation of a polyphenol–pepsin complex through mechanisms such as hydrogen bonding and van der Waals interactions, along with a decrease in the hydrolysis rate induced by pancreatin [34,35]. Tomás-Barberán [36] indicated that the restricted absorption of dietary polyphenols in the gastrointestinal tract results from their resistance to gastrointestinal and absorptive processes. This is because the majority of polyphenols, along with nondigestible carbohydrates and other plant components like lignin, resistant proteins, and carotenoids, display a low sensitivity to these physiological mechanisms.

### 3.2. Impact of ACOD on Pathogenic and Probiotic Bacteria

**Figure 1:**
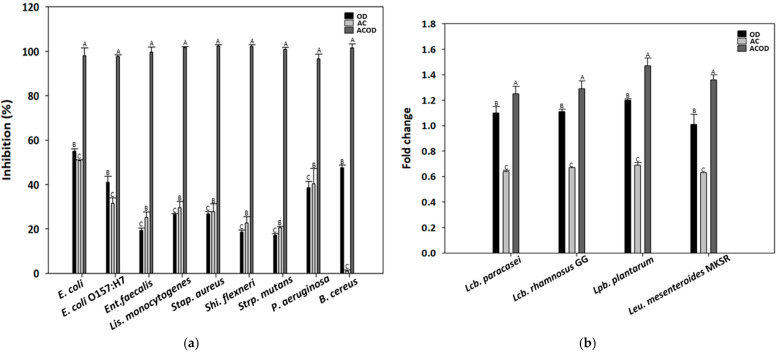
Differential growth of bacterial strains on ACOD, OD, and AC. (**a**) Inhibition rate of pathogenic bacteria growth by ACOD, OD, and AC; (**b**) Fold change of probiotic bacteria growth by ACOD, OD, and AC. ACOD is a transglycosylation product of sucrose and maltose in the presence of *Artemisia capillaris* catalyzed by *Leu. mesenteroides* MKSR dextransucrase; OD is a transglycosylation product of sucrose and maltose catalyzed by MKSR dextransucrase; AC is hot water extracted *A. capillaris*. All values are mean ± standard deviation (*n* = 3). Different capital letters are significantly different (*p* < 0.05).

Figure 1 shows the relative growth-promoting ratios of ACOD in selected bacterial strains in pure culture. An *A. capillaris*-derived transglycosylated product significantly inhibited the growth of nine pathogenic bacteria, which are associated with various diseases and foodborne poisoning incidences. However, ACOD significantly enhanced the growth of probiotic strains like *Lcb. rhamnosus*, *Lcb. paracasei*, *Lpb. plantarum*, and *Leu. mesenteroides* MKSR. Notably, probiotics are living organisms, not chemical compounds. Their health benefits may arise from various activities, including interactions with the human host and its gut microbiota. Specifically, probiotic health-promoting mechanisms include: (i) strengthening epithelial barrier function, (ii) modulating the gut microbiota, (iii) competitively excluding and inhibiting pathogens, (iv) producing SCFAs (considered a common mechanism shared among strains), (v) generating other small compounds with systemic activity, and (vi) modulating the immune system with various strain-specific diverse activities [37,38]. Consequently, the positive impact on health can be an indirect result of various biological activities, and pinpointing the exact mechanism responsible for a clinical outcome can be challenging [38].

Several studies have reported that each bacterial strain exhibits different carbohydrate utilization abilities [39]. For example, Hu et al. [40] found that *Lactobacillus* species preferentially metabolizes short-chain oligosaccharides like IMOs. The selective utilization of IMOs by probiotic strains such as *Bif. longum* TISTR 2195 and *Lpb. plantarum* TISTR 1465 was also demonstrated in the study by Tiangpook et al. [41], which showed that IMOs did not facilitate the growth of the pathogenic bacterium *E. coli* TISTR 117. Fuhren et al. [42] reported that 77 *Lpb. plantarum* strains have diverse and strain-specific abilities to utilize various prebiotic fibers. This strain-specific promotion of growth via prebiotic substrates has the potential to increase the persistence and activity of *Lpb. plantarum* in the gut. Wei et al. [5] focused their research on high-molecular-weight prebiotics, noting that these prebiotic substances can persist in the colon and reach the farthest region of the intestinal tract, which is the origin of many chronic diseases. For instance, dextran has been shown to promote the growth of probiotics such as *Bif. lactis*, *Bif. infantis*, *Lactobacillus acidophilus*, and *Lcb. casei* while being resistance to utilization by pathogenic bacteria like *Clostridium difficile*, *C. butyricum*, *B. cereus*, *E. coli*, and *Salmonella* Typhimurium [43,44].

The health beneficial effects of Lactobacillus species are well-documented, including the production of short-chain fatty acids (SCFAs). The Korean Ministry of Food and Drug Safety has approved 19 *Lactobacillus* species for use as probiotics. *Leu. mesenteroides* MKSR has particularly shown substantial probiotic potential, with antidiabetic, antioxidant, and cholesterol-lowering effects [45]. Our previous research further demonstrated that intervention with *Momordica charantia* fermented by *Leu. mesenteroides* MKSR significantly improved metabolic health by regulating blood sugar and cholesterol levels and reduced body fat in high-fat and high-cholesterol diet-induced obese animal models [31].

Meanwhile, the positive effect of ACOD on the suppression of harmful bacteria is not only the role of IMOs and dextran but also that of *A. capillaris*. Several research investigations have documented the antibacterial properties of *Artemisia* species against *B. cereus*, *Stap. Aureus*, *E. coli*, *Klebsiella pneumoniae*, *Lis. Monocytogenes*, and even probiotics such as *Lpb. Plantarum* and *Leu. Mesenteroides* [46,47,48,49]. The antibacterial activity of AC depends on the extraction solvent and concentration and is potentially attributable to compounds such as capillin, scopoletin, and escoparone, which have been isolated from *Artemisia* spp. [50,51].

### 3.3. Effect of ACOD on SCFAs Production during Fecal Fermentation

SCFAs, which are the main metabolites produced by the gut microbiota and include acetic, lactic, propionic, *n*-butyric, *i*-butyric, *n*-valeric, and *i*-valeric acids, are influenced by both the host’s diet and the symbiotic microbiota in the gut. Furthermore, SCFAs play a crucial role in preserving the normal function of the gut, influencing the morphology and function of colonic epithelial cells, promoting sodium absorption, and serving as energy sources for various tissues [21]. They also contribute to regulating appetite, reducing cholesterol levels, and enhancing insulin sensitivity [21,52,53,54]. Although SCFAs offer many advantages, they may also cause some undesirable effects. The fermentation process that generates SCFAs often results in gas production like hydrogen, methane, and carbon dioxide, which can cause discomfort, bloating, and flatulence in some individuals [55,56]. The severity of these can be highly variable and depend on individual gut microbiota composition and the types of fibers or prebiotics consumed. Therefore, while the overall health benefits of SCFAs generally surpass these drawbacks, their potential to cause digestive discomfort should not be overlooked in dietary interventions aimed at modulating gut microbiota. After 24 h-fecal fermentation, lactic acid, acetic acid, propionic acid, and *n*-butyrate were predominantly produced in the blank, FOS, and ACOD groups (Table 2). Notably, the lactic acid concentration in the ACOD group (31.30 ± 0.59 mM) was significantly higher than the FOS group (7.12 ± 2.54 mM) and the blank group (0.07 ± 0.02 mM). Bourriaud et al. [57] reported that an increase in lactic acid levels leads to the synthesis of butyric acid within the gut microbiota, and certain bacteria utilize lactic acid for their growth. The activities of these lactate-utilizing bacteria play a crucial role in determining lactate concentrations [58]. Notably, certain species within the Firmicutes phylum are significant lactate utilizers, capable of producing beneficial SCFAs like butyrate or propionate from lactate [52,53]. As these SCFAs have known positive effects on the host, the utilization of lactate can be seen as an indirectly beneficial outcome of lactate production by other members of the gut microbiota. In our experiments, the conversion of lactic acid to butyric acid can also be expected [57]. The concentrations of acetic and propionic acids in the ACOD group were much higher than those of the blank and FOS groups. Acetic acid is not only one of the main end products of fermentation of *Bifidobacterium* and *Lactobacillus*, but also the source of energy for the brain, heart, and peripheral tissues [54]. In particular, it is able to traverse the blood–brain barrier to suppress appetite through a central homeostatic mechanism [55]. The increase in acetic acid levels is because many enteric bacteria, including *Bacteroides*, *Prevotella*, and *Bifidobacterium*, produce acetic acid from pyruvate through the acetyl-CoA pathway [26]. Propionic acid, when present in the intestinal tract, undergoes metabolism in the liver, where it serves as a glucogenic substrate and inhibits cholesterol synthesis [59]. At the same time, propionic acid, although metabolized in the liver, exists in low concentrations in the periphery, making acetate the most abundant SCFA in the peripheral circulation [60]. Furthermore, propionic acid has the potential to reduce serum cholesterol levels, protect against diet-induced obesity, and improve tissue insulin sensitivity [61]. The concentrations of *n*-butyric acid in the FOS (3.21 ± 1.52 mM) and ACOD groups (3.91 ± 1.70 mM) were significantly higher than the blank group (0.20 ± 0.14 mM). Butyric acid serves as an energy source for intestinal epithelial cells and maintains the integrity of the intestinal barrier, thereby enhancing intestinal immunity, while other absorbed SCFAs drain into the portal vein [21,55]. The high concentration of *n*-butyric acid in the FOS and ACOD groups is probably due to the relative abundance of Actinobacteria in the FOS group and Firmicutes in the ACOD group [62]. Finally, the concentration of *i*-butyric, *i*-valeric, and *n*-valeric acids was found to be extremely low or negligible, which could be associated with a small number of specific bacteria [63].

### 3.4. Effect of ACOD on the Gut Microbiota

The human gut microbiota is essential for human health, especially affecting energy metabolism and the immune system [64]. Prebiotics, fermented by gut microorganisms, are a useful dietary source that provides health benefits to the host by influencing the gut microbiota. Understanding the relationship between prebiotic and the gut microbiota is important to preventing diseases and promoting health by regulating intestinal bacteria.

**Figure 2:**
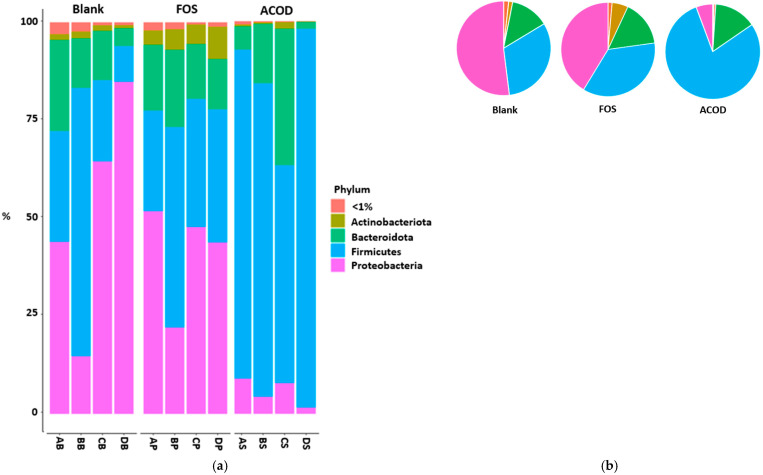
Taxonomic composition of gut microbiota during in vitro fermentation. (**a**) Stacked bar plot shows the relative abundance at the phylum level; (**b**) Pie chart shows the average relative abundance at the phylum level. AB~DB, The blank control (no additional carbon source supplement); AP~DP, the positive control (the fructooligosaccharides (FOS) supplement); AS~DS, the experimental group (the *Artemisia capillaris*-derived transglycosylated product (ACOD) supplement).

The relative abundance in the gut microbiota is shown in Figure 2. The gut microbiota of a healthy adult encompasses over a thousand different bacterial species. Although more than 90% of these bacteria at the phylum level belong to the categories Firmicutes and Bacteroides, a significant presence of bacteria of the phyla Proteobacteria, Actinobacteria, Verrucomicrobia, Fusobacteria, and Cyanobacteria is also observed in the human intestinal tract [65]. At the phylum level, the dominant bacteria of the blank group were identified as Proteobacteria (51.96%), Firmicutes (31.68%), and Bacteroidetes (13.25%). Compared to the blank group, a significant increase in Firmicutes (78.90%) and a significant decrease in Proteobacteria (5.68%) were found in the ACOD group. There were no significant differences in Bacteroidetes among the three groups. Previous research indicated that an interaction between Firmicutes and Bacteroidetes is associated with the maintenance of homeostasis [66]. Furthermore, alterations in this ratio have been linked to the development of various pathologies. For example, elevated levels of Firmicutes have been linked to obesity, while increased levels of Bacteroidetes have been associated with intestinal inflammation [67]. Although the F/B ratio receives significant attention, it is important to remember which genus influenced the change in the ratio since beneficial bacteria such as *Lactobacillus*, which are classified mainly as probiotics, also belong to Firmicutes. Research examining key Firmicutes and Bacteroidetes species linked to obesity demonstrated a robust correlation between obesity and specific Firmicutes bacteria, namely *Blautia hydrogenotrophica*, *Coprococcus catus*, *Eubacterium ventriosum*, *Ruminococcus bromii*, and *Ruminococcus obeum*. Proteobacteria, recognized as the most variable phylum, are commonly found in the gut microbiota of healthy individuals and include well-known pathogens such as *Escherichia coli*, *Shigella*, *Salmonella*, and *Campylobacter*. Proteobacteria are associated with dysbiosis and are linked to a reduction in *Firmicutes* and overall microbial diversity in inflammatory bowel disease [63,68]. The relative abundance of Proteobacteria significantly decreased in the ACOD group, indicating a reduction in conditional pathogen [69]. The gut microbiome changes in the FOS group differed from the ACOD group. Compared to the blank group, there was no significant difference in the relative abundance of Firmicutes (35.90%) and Proteobacteria (41.31%). However, there was a significant increase in the relative abundance of Actinobacteria (5.54%) in the FOS group. Actinobacteria, particularly *Bifidobacterium*, play an important role in promoting gut health. Dou et al. [70] and Shadid et al. [71] reported that FOSs induced *Bifidobacterium* proliferation in the intestine. These results indicate that both ACOD and FOSs can alter the composition of the gut microbiome, particularly by promoting the proliferation of beneficial bacteria. However, their effects on the regulation of gut microbiome differ significantly.

**Figure 3:**
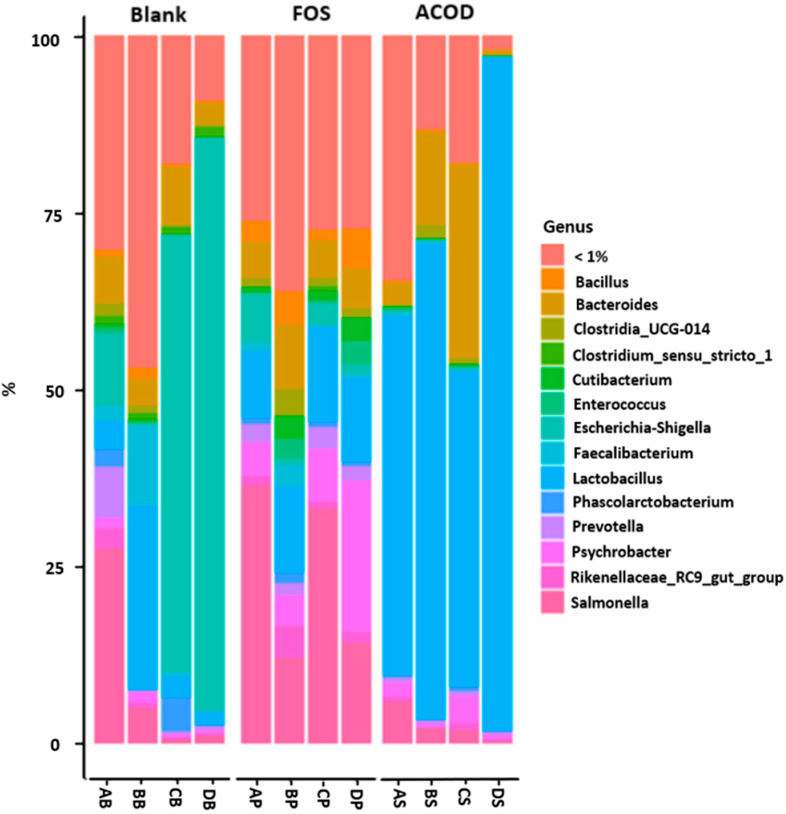
The relative abundance of the bacterial community at the genus level. AB~DB, The blank control (no additional carbon source supplement); AP~DP, the positive control (the fructooligosaccharides (FOS) supplement); AS~DS, the experimental group (the *Artemisia capillaris*-derived transglycosylated product (ACOD) supplement).

At the genus level (Figure 3), the blank group was mainly composed of *Escherichia-Shigella* (38.28%), *Lactobacillus* (8.76%), *Salmonella* (8.60%) and *Bacteroides* (5.41%). Compared to the blank group, an increase in *Lactobacillus* (64.44%) was found in the ACOD group. *Lactobacillus* can exert health benefits in several ways, for example, by inhibiting the growth of pathogenic bacteria as they produce lactic, propionic, and acetic acids, lowering the pH that suppresses the proliferation of pathogens in the intestinal tract. Furthermore, they can competitively prevent the attachment of pathogenic bacteria to the epithelium [72]. Importantly, key probiotic bacteria in the gut microbiota are members of the *Lactobacillus* genus classified under the Firmicutes phylum [73]. The health-promoting effects of probiotics are the result of intricate and sometimes overlapping mechanisms. These mechanisms are typically specific to the probiotic strain, although some aspects may be shared among strains within the same taxonomic group, such as the *Lactobacillus* genus [74]. Heeney et al. [75] explored the potential beneficial role of the *Lactobacillus* genus in the intestine and in the context of treatment with probiotics. Interestingly, in the current study, *Escherichia/Shigella* was significantly decreased in the ACOD group (0.22%) compared to the blank group (38.28%). *Escherichia/Shigella* is one of the pathogens in the human gut that could affect host health and cause unstable gut microbiota associated with low-grade inflammation or chronic colitis [63]. Then, ACOD becomes the carbon source of *Lactobacillus* and induces proliferation, leading to *Lactobacillus* growth and suppressing harmful bacteria such as *Escherichia*/*Shigella*. This is the same as the effect on the individual culture of Figure 1. Similarly, *Escherichia/Shigella* was also significantly decreased in the FOS group (3.13%) compared to the blank group (38.28%). This result might be because *Escherichia/Shigella* cannot use oligosaccharides like FOSs as it is deficient in carbohydrate-active enzymes [76]. However, in the FOS group, *Psychrobacter* and *Salmonella* increased significantly compared to the blank group. *Salmonella* has been extensively researched as a pathogen and is recognized for its ability to establish a specific environment in the intestinal tract by inducing inflammation, modifying the composition of the intestinal microbiota, and using nutrients produced by the intestinal microbiota [77]. According to Bridier et al. [78], *Psychrobacter* can contribute to the spread of antimicrobial resistance through interaction with pathogens in pigs. Some studies have reported that FOSs increase the number of *Salmonella* according to the FOS dose [79,80]. Mao et al. [81] reported that after 3 weeks, Firmicutes abundance remained at 60% for the FOS-low group but dropped to 35% for the FOS-high group, indicating that the effect of FOSs on the composition of the intestinal microbiota was dependent on the dose. Furthermore, Petersen et al. [82] reported that the addition of 10% FOSs to a corn-based rodent diet increased *Salmonella* translocation. According to Kamada et al. [76], *Salmonella* exploits intestinal inflammation induced by the pathogen itself. In the intestinal environment, commensal microorganisms produce a significant amount of hydrogen sulfide (H_2_S), and the epithelium transforms H_2_S into thiosulfate (S_2_O_3_^2−^) to prevent the H_2_S-related toxic effects related to H_2_S in host cells. *Salmonella* infection leads to the recruitment of neutrophils that generate reactive oxygen species, causing the conversion of S_2_O_3_^2−^ into tetrathionate (S_4_O_6_^2−^). Unlike commensals, *Salmonella* possesses the ttrSR ttrBCA operon, enabling the use of S_4_O_6_^2−^. This confers a growth advantage to *Salmonella* over commensal microbes in an inflamed environment. Furthermore, S_4_O_6_^2−^ supports the anaerobic growth of *Salmonella* on ethanolamine. Furthermore, FOSs are generally hydrolyzed by 32 enzymes of the glycoside hydrolase (GH) family, and the resultant fructose and glucose are metabolized using indigenous metabolic pathways in each microbe [83]. However, *Salmonella* does not have the GH32 enzyme, so it cannot directly degrade and use FOSs. Endo et al. [84] characterized extracellular GH32 enzymes in the organisms to consider possible cross-feeding of FOSs with other microbes. In the present study, *Bacillus subtilis*, which is known to produce extracellular GH32 enzymes [85], increased significantly in the FOS group (3.66%) compared to the blank group (0.86%) and the ACOD group (0.23%). This may have affected the cross-feeding of *Salmonella* and thus its proliferation. Although FOSs help butyrate production by increasing Actinobacteria as a prebiotic, it may possess a limit in increasing *Salmonella* depending on the dose. The presence or absence of AC can also affect *Salmonella* growth in the FOS group. Ahameethunisa and Hopper [86] reported that one of the *Artemisia spp*., *Artemisia nilagirica,* inhibited the growth of *Salmonella*. Das et al. [87] reported that cranberry pomace and the flavonol-rich fraction had bactericidal effects against *Salmonella* strains. Similarly, AC’s influence cannot be ignored. Studies indicate that a significant portion of dietary polyphenols is not absorbed in the small intestine. Instead, these unabsorbed components accumulate in the large intestine, where they undergo extensive metabolism by the gut microbiota [88]. The polyphenols in the diet affect the composition of the gut microbiota in the host, thereby influencing the host’s metabolism. Concurrently, the intestinal microbiota has the ability to metabolize polyphenols into small phenolic metabolites, thus influencing the regulatory metabolic network.

Overall, ACOD improved the gut microbiota by increasing the relative abundance of *Lactobacillus* while reducing *Escherichia*/*Shigella*, indicating a positive effect on beneficial bacteria and the suppression of harmful pathogenic bacteria. In contrast, FOSs showed mixed effects, promoting both beneficial bacteria and potentially harmful pathogens like *Salmonella*. Future research should explore the dose-dependent effects of ACOD and its long-term health implication.

## 4. Conclusions

This study demonstrated that ACOD is highly fermentable by the human gut microbiota, leading to substantial production of SCFAs, particularly lactic acid, acetic acid, propionic acid, and butyric acid. The ability of ACOD to enhance SCFA production indicates that it is effectively utilized by intestinal microorganisms as a source of energy, particularly due to its plant fiber and undigestible carbohydrates. Furthermore, ACOD significantly modulated the composition of the gut microbiota by promoting the growth of beneficial bacteria, such as *Lactobacillus* species, while simultaneously reducing the abundance of pathogenic bacteria, including *Escherichia/Shigella*. These findings highlight the potential of ACOD as a prebiotic candidate and functional food additive that not only supports the growth of probiotics but also improves overall gut health by inhibiting the growth of harmful bacteria. This study contributes to the broader understanding of prebiotics and their impact on gut microbiota. The distinct microbial modulation by ACOD, compared to fructooligosaccharides (FOSs), underscores its potential as a novel prebiotic with promising applications in both food and pharmaceutical industries. However, further research is needed to explore the long-term effects, optimal dosage, and safety of ACOD through clinical trials. Moreover, mechanistic studies should investigate how ACOD interacts with specific gut bacteria and its role in driving health benefits, ultimately enabling its effective application in human health.

## Figures and Tables

**Table 1:** Total carbohydrate, polyphenol, and flavonoid contents of ACOD before and after in vitro digestion.

	ACOD		Remaining (%)
	Before Digestion	After Digestion	
Total carbohydrate (mg/g)	1284.2 ± 16.86	1284.2 ± 69.35	100% ± 5.40
Total polyphenol contents (mgGAE/g)	24.2 ± 0.11	21.1 ± 0.02	87.19% ± 0.09
Total flavonoid contents (mgQE/g)	8.7 ± 0.01	7.20 ± 0.06	82.76% ± 0.90

**Table 2:** Short-chain fatty acid concentration (mM) during in vitro fermentation by human fecal microbiota.

	Acetic Acid	Propionic Acid	*n*-Butyric Acid	Lactic Acid	*i*-Butyric Acid	*i*-Valeric Acid	*n*-Valeric Acid
Blank	2.27 ± 0.18 ^C^	0.002 ± 0.002 ^C^	0.002 ± 0.002 ^B^	0.07 ± 0.02 ^C^	0.0004 ± 0.00 ^B^	0.0002 ± 0.00 ^C^	0.00 ± 0.00 ^B^
FOS	3.30 ± 0.49 ^B^	0.035 ± 0.023 ^B^	0.036 ± 0.017 ^A^	7.12 ± 2.54 ^B^	0.01 ± 0.00 ^A^	0.0037 ± 0.00 ^B^	0.01 ± 0.01 ^A^
ACOD	14.72 ± 0.31 ^A^	0.115 ± 0.04 ^A^	0.044 ± 0.019 ^A^	31.30 ± 0.59 ^A^	0.01 ± 0.00 ^A^	0.0050 ± 0.00 ^A^	0.01 ± 0.01 ^A^

Data are shown as mean ± standard deviation (*n* = 3). Different capital letters indicate significantly different concentration between groups (*p* < 0.05).

## Data Availability

The data presented in this study are available on request from the corresponding author.

## References

[1] Hou K., Wu Z.X., Chen X.Y., Wang J.Q., Zhang D., Xiao C., Chen Z.S. (2022). Microbiota in health and diseases. Signal Transduct. Target. Ther..

[2] Rawi M.H., Zaman S.A., Pa’ee K.F., Leong S.S., Sarbini S.R. (2020). Prebiotics metabolism by gut-isolated probiotics. J. Food Sci. Technol..

[3] Peredo-Lovillo A., Romero-Luna H.E., Jiménez-Fernández M. (2020). Health promoting microbial metabolites produced by gut microbiota after prebiotics metabolism. Food Res. Int..

[4] Nogacka A.M., Salazar N., Arboleya S., Ruas-Madiedo P., Mancabelli L., Suarez A., Gueimonde M. (2020). In vitro evaluation of different prebiotics on the modulation of gut microbiota composition and function in morbid obese and normal-weight subjects. Int. J. Mol. Sci..

[5] Wei B., Xia W., Wang L., Jin X., Yang W., Rao D., Wu J. (2022). Diverse prebiotic effects of isomaltodextrins with different glycosidic linkages and molecular weights on human gut bacteria in vitro. Carbohydr. Polym..

[6] Banerjee A., Somasundaram I., Das D., Jain M.S., Banu H., Mitta S.P., Pathak S. (2023). Functional foods: A promising strategy for restoring gut microbiota diversity impacted by SARS-CoV-2 Variants. Nutrients.

[7] Cunningham M., Azcarate-Peril M.A., Barnard A., Benoit V., Grimaldi R., Guyonnet D., Gibson G.R. (2021). Shaping the future of probiotics and prebiotics. Trends Microbiol..

[8] Bindels L.B., Delzenne N.M., Cani P.D., Walter J. (2015). Towards a more comprehensive concept for prebiotics. Nat. Rev. Gastroenterol. Hepatol..

[9] Lee S., Park J., Jang J.K., Lee B.H., Park Y.S. (2019). Structural analysis of gluco-oligosaccharides produced by *Leuconostoc lactis* and their prebiotic effect. Molecules.

[10] Scott K.P., Grimaldi R., Cunningham M., Sarbini S.R., Wijeyesekera A., Tang M.L., Gibson G.R. (2020). Developments in understanding and applying prebiotics in research and practice—An ISAPP conference paper. J. Appl. Microbiol..

[11] Gibson G.R., Hutkins R., Sanders M.E., Prescott S.L., Reimer R.A., Salminen S.J., Reid G. (2017). Expert consensus document: The International Scientific Association for Probiotics and Prebiotics (ISAPP) consensus statement on the definition and scope of prebiotics. Nat. Rev. Gastroenterol. Hepatol..

[12] Wang X., Qi Y., Zheng H. (2022). Dietary polyphenol, gut microbiota, and health benefits. Antioxidants.

[13] Rodríguez-Daza M.C., Pulido-Mateos E.C., Lupien-Meilleur J., Guyonnet D., Desjardins Y., Roy D. (2021). Polyphenol-mediated gut microbiota modulation: Toward prebiotics and further. Front. Nutr..

[14] Yoon J., Kim M. (2022). In vitro evaluation of antidiabetic, antidementia, and antioxidant activity of Artemisia capillaris fermented by *Leuconostoc* spp.. LWT.

[15] Hsueh T.P., Lin W.L., Dalley J.W., Tsai T.H. (2021). The pharmacological effects and pharmacokinetics of active compounds of *Artemisia capillaris*. Biomedicines.

[16] Bisht D., Kumar D., Kumar D., Dua K., Chellappan D.K. (2021). Phytochemistry and pharmacological activity of the genus *Artemisia*. Arch. Pharm. Res..

[17] Méndez-Líter J.A., Tundidor I., Nieto-Domínguez M., de Toro B.F., González Santana A., de Eugenio L.I., Martínez M.J. (2019). Transglycosylation products generated by *Talaromyces amestolkiae* GH3 β-glucosidases: Effect of hydroxytyrosol, vanillin and its glucosides on breast cancer cells. Microb. Cell Factories.

[18] Kim G.E., Kang H.K., Seo E.S., Jung S.H., Park J.S., Kim D.H., Kim D. (2012). Glucosylation of the flavonoid, astragalin by *Leuconostoc mesenteroides* B-512FMCM dextransucrase acceptor reactions and characterization of the products. Enzyme Microb. Technol..

[19] Moon H., Kim M. (2024). Enzymatic synthesis of *Artemisia capillaris*-derived prebiotic oligosaccharide using *Leuconostoc mesenteroides* MKSR dextransucrase and its potential health functional effects. Food Biosci..

[20] Guo Y., Chen X., Gong P., Li G., Yao W., Yang W. (2023). The gut–organ-axis concept: Advances the application of gut-on-chip technology. Int. J. Mol. Sci..

[21] Ding Y., Yan Y., Peng Y., Chen D., Mi J., Lu L., Cao Y. (2019). In vitro digestion under simulated saliva, gastric and small intestinal conditions and fermentation by human gut microbiota of polysaccharides from the fruits of *Lycium barbarum*. Int. J. Biol. Macromol..

[22] Hwang E.S., Kim S. (2023). Effect of in vitro gastrointestinal digestion on phytochemicals and antioxidant activities in cherry tomatoes (*Solanum lycopersicum* var. cerasiforme). Prev. Nutr. Food Sci..

[23] DuBois M., Gilles K.A., Hamilton J.K., Rebers P.T., Smith F. (1956). Colorimetric method for determination of sugars and related substances. Anal. Chem..

[24] Chen Y., Lin H., Lin M., Zheng Y., Chen J. (2020). Effect of roasting and in vitro digestion on phenolic profiles and antioxidant activity of water-soluble extracts from sesame. Food Chem. Toxicol..

[25] Vijayakumar P.P., Muriana P.M. (2015). A microplate growth inhibition assay for screening bacteriocins against *Listeria monocytogenes* to differentiate their mode of action. Biomolecules.

[26] Hajar-Azhari S., Abd Rahim M.H., Sarbini S.R., Muhialdin B.J., Olusegun L., Saari N. (2021). Enzymatically synthesized fructooligosaccharides from sugarcane syrup modulate the composition and short-chain fatty acid production of the human intestinal microbiota. Food Res. Int..

[27] Callahan B.J., McMurdie P.J., Rosen M.J., Han A.W., Johnson A.J.A., Holmes S.P. (2016). DADA2: High-resolution sample inference from Illumina amplicon data. Nat. Methods.

[28] Martin M. (2011). Cutadapt removes adapter sequences from high-throughput sequencing reads. EMBnet. J..

[29] Song W.S., Park H.G., Kim S.M., Jo S.H., Kim B.G., Theberge A.B., Kim Y.G. (2020). Chemical derivatization-based LC–MS/MS method for quantitation of gut microbial short-chain fatty acids. J. Ind. Eng. Chem..

[30] Davani-Davari D., Negahdaripour M., Karimzadeh I., Seifan M., Mohkam M., Masoumi S.J., Ghasemi Y. (2019). Prebiotics: Definition, types, sources, mechanisms, and clinical applications. Foods.

[31] Moon H., Ha J.H., Lee J., Jang H., Kwon D., Cho M., Kim M. (2023). The effect of fermented *Momordica charantia* with *Leuconostoc mesenteroides* MKSR on metabolic complications induced by high-fat high-cholesterol diet in C57BL/6 mice. Fermentation.

[32] Zhang T., Yang Y., Liang Y., Jiao X., Zhao C. (2018). Beneficial effect of intestinal fermentation of natural polysaccharides. Nutrients.

[33] Lima K., Silva O., Figueira M.E., Pires C., Cruz D., Gomes S., Duarte M.P. (2019). Influence of the in vitro gastrointestinal digestion on the antioxidant activity of *Artemisia gorgonum Webb* and *Hyptis pectinata* (L.) *Poit*. infusions from Cape Verde. Food Res. Int..

[34] Zhao Q., Wang Z., Wang X., Yan X., Guo Q., Yue Y., Yuan Y. (2023). The bioaccessibility, bioavailability, bioactivity, and prebiotic effects of phenolic compounds from raw and solid-fermented mulberry leaves during in vitro digestion and colonic fermentation. Food Res. Int..

[35] Zhou H., Hu Y., Tan Y., Zhang Z., McClements D.J. (2021). Digestibility and gastrointestinal fate of meat versus plant-based meat analogs: An in vitro comparison. Food Chem..

[36] Tomás-Barberán F.A., Selma M.V., Espín J.C. (2016). Interactions of gut microbiota with dietary polyphenols and consequences to human health. Curr. Opin. Clin. Nutr. Metab. Care.

[37] Medoro A., Davinelli S., Colletti A., Di Micoli V., Grandi E., Fogacci F., Cicero A.F.G. (2023). Nutraceuticals as modulators of immune function: A review of potential therapeutic effects. Prev. Nutr. Food Sci..

[38] De Bellis P., Sisto A., Lavermicocca P. (2021). Probiotic bacteria and plant-based matrices: An association with improved health-promoting features. J. Funct. Foods.

[39] Solopova A., van Sinderen D., van Sinderen D., Ventura M. (2021). Determination of bifidobacterial carbohydrate utilization abilities and associated metabolic end products. Bifidobacteria.

[40] Hu Y., Ketabi A., Buchko A., Gänzle M.G. (2013). Metabolism of isomalto-oligosaccharides by *Lactobacillus reuteri* and *bifidobacteria*. Lett. Appl. Microbiol..

[41] Tiangpook S., Nhim S., Prangthip P., Pason P., Tachaapaikoon C., Ratanakhanokchai K., Waeonukul R. (2023). Production of a series of long-chain isomaltooligosaccharides from maltose by *Bacillus subtilis* AP-1 and associated prebiotic properties. Foods.

[42] Fuhren J., Rösch C., Ten Napel M., Schols H.A., Kleerebezem M. (2020). Synbiotic matchmaking in *Lactobacillus plantarum*: Substrate screening and gene-trait matching to characterize strain-specific carbohydrate utilization. Appl. Environ. Microbiol..

[43] Kim G., Bae J.H., Cheon S., Lee D.H., Kim D.H., Lee D., Han N.S. (2022). Prebiotic activities of dextran from *Leuconostoc mesenteroides* SPCL742 analyzed in the aspect of the human gut microbial ecosystem. Food Funct..

[44] Tingirikari J.M.R., Kothari D., Goyal A. (2014). Superior prebiotic and physicochemical properties of novel dextran from *Weissella cibaria* JAG8 for potential food applications. Food Funct..

[45] Lee S., Kim M. (2019). *Leuconostoc mesenteroides* MKSR isolated from kimchi possesses α-glucosidase inhibitory activity, antioxidant activity, and cholesterol-lowering effects. LWT.

[46] Isshiki K., Nishinomiya T., Nozaka N., Tokuka K. (1993). Growth inhibition of microorganisms by plant extracts. Nippon Shokuhin Kogyo Gakkaishi.

[47] Sagdic O., Karahan A.G., Ozcan M., Ozkan G. (2003). Note: Effect of some spice extracts on bacterial inhibition. Food Sci. Technol. Int..

[48] Naili M.B., Alghazeer R.O., Saleh N.A., Al-Najjar A.Y. (2010). Evaluation of antibacterial and antioxidant activities of *Artemisia campestris* (Astraceae) and *Ziziphus lotus* (Rhamnaceae). Arab. J. Chem..

[49] Mannan H.A., Ahmed I.B.R.A.R., Hussain I.Z.H.A.R., Jamil M., Miza B. (2012). Antibacterial activity and brine shrimp toxicity of *Artemisia dubia* extract. Pak. J. Bot..

[50] Ghlissi Z., Sayari N., Kallel R., Bougatef A., Sahnoun Z. (2016). Antioxidant, antibacterial, anti-inflammatory and wound healing effects of *Artemisia campestris* aqueous extract in rat. Biomed. Pharmacother..

[51] Bora K.S., Sharma A. (2011). The genus Artemisia: A comprehensive review. Pharm. Biol..

[52] Duncan S.H., Louis P., Flint H.J. (2004). Lactate-utilizing bacteria, isolated from human feces, that produce butyrate as a major fermentation product. Appl. Environ. Microbiol..

[53] Reichardt N., Duncan S.H., Young P., Belenguer A., McWilliam Leitch C., Scott K.P., Louis P. (2014). Phylogenetic distribution of three pathways for propionate production within the human gut microbiota. ISME J..

[54] Wu D.T., Fu Y., Guo H., Yuan Q., Nie X.R., Wang S.P., Gan R.Y. (2021). In vitro simulated digestion and fecal fermentation of polysaccharides from loquat leaves: Dynamic changes in physicochemical properties and impacts on human gut microbiota. Int. J. Biol. Macromol..

[55] Wu D.T., Nie X.R., Gan R.Y., Guo H., Fu Y., Yuan Q., Qin W. (2021). In vitro digestion and fecal fermentation behaviors of a pectic polysaccharide from okra (*Abelmoschus esculentus*) and its impacts on human gut microbiota. Food Hydrocoll..

[56] Cummings J.H., Macfarlane G.T. (1991). The control and consequences of bacterial fermentation in the human colon. J. Appl. Bacteriol..

[57] Bourriaud C., Robins R.J., Martin L., Kozlowski F., Tenailleau E., Cherbut C., Michel C. (2005). Lactate is mainly fermented to butyrate by human intestinal microflora, but inter-individual variation is evident. J. Appl. Microbiol..

[58] Wang S.P., Rubio L.A., Duncan S.H., Donachie G.E., Holtrop G., Lo G., Flint H.J. (2020). Pivotal roles for pH, lactate, and lactate-utilizing bacteria in the stability of a human colonic microbial ecosystem. mSystems.

[59] Venter C.S., Vorster H.H., Cummings J.H. (1990). Effects of dietary propionate on carbohydrate and lipid metabolism in healthy volunteers. Am. J. Gastroenterol..

[60] Wang M., Wichienchot S., He X., Fu X., Huang Q., Zhang B. (2019). In vitro colonic fermentation of dietary fibers: Fermentation rate, short-chain fatty acid production, and changes in microbiota. Trends Food Sci. Technol..

[61] Fu Y., Zhang J., Chen K., Xiao C., Fan L., Zhang B., Fang B. (2019). An in vitro fermentation study on the effects of *Dendrobium officinale* polysaccharides on human intestinal microbiota from fecal microbiota transplantation donors. J. Funct. Foods.

[62] Fu X., Liu Z., Zhu C., Mou H., Kong Q. (2019). Nondigestible carbohydrates, butyrate, and butyrate-producing bacteria. Crit. Rev. Food Sci. Nutr..

[63] Zhang X., Aweya J.J., Huang Z.X., Kang Z.Y., Bai Z.H., Li K.H., Cheong K.L. (2020). In vitro fermentation of *Gracilaria lemaneiformis* sulfated polysaccharides and its agaro-oligosaccharides by human fecal inocula and its impact on microbiota. Carbohydr. Polym..

[64] Afzaal M., Saeed F., Shah Y.A., Hussain M., Rabail R., Socol C.T., Aadil R.M. (2022). Human gut microbiota in health and disease: Unveiling the relationship. Front. Microbiol..

[65] Chen D., Chen G., Chen C., Zeng X., Ye H. (2020). Prebiotics effects in vitro of polysaccharides from tea flowers on gut microbiota of healthy persons and patients with inflammatory bowel disease. Int. J. Biol. Macromol..

[66] Magne F., Gotteland M., Gauthier L., Zazueta A., Pesoa S., Navarrete P., Balamurugan R. (2020). The firmicutes/bacteroidetes ratio: A relevant marker of gut dysbiosis in obese patients?. Nutrients.

[67] Tseng C.H., Wu C.Y. (2019). The gut microbiome in obesity. J. Formos. Med. Assoc..

[68] Stojanov S., Berlec A., Štrukelj B. (2020). The influence of probiotics on the Firmicutes/Bacteroidetes ratio in the treatment of obesity and inflammatory bowel disease. Microorganisms.

[69] Xu S.Y., Chen X.Q., Liu Y., Cheong K.L. (2020). Ultrasonic/microwave-assisted extraction, simulated digestion, and fermentation in vitro by human intestinal flora of polysaccharides from *Porphyra haitanensis*. Int. J. Biol. Macromol..

[70] Dou Y., Yu X., Luo Y., Chen B., Ma D., Zhu J. (2022). Effect of fructooligosaccharides supplementation on the gut microbiota in humans: A systematic review and meta-analysis. Nutrients.

[71] Shadid R., Haarman M., Knol J., Theis W., Beermann C., Rjosk-Dendorfer D., Krauss-Etschmann S. (2007). Effects of galactooligosaccharide and long-chain fructooligosaccharide supplementation during pregnancy on maternal and neonatal microbiota and immunity—A randomized, double-blind, placebo-controlled study. Am. J. Clin. Nutr..

[72] Celebioglu H.U., Svensson B. (2018). Dietary nutrients, proteomes, and adhesion of probiotic *Lactobacilli* to mucin and host epithelial cells. Microorganisms.

[73] Rastogi S., Singh A. (2022). Gut microbiome and human health: Exploring how the probiotic genus *Lactobacillus* modulates immune responses. Front. Pharmacol..

[74] Campana R., van Hemert S., Baffone W. (2017). Strain-specific probiotic properties of lactic acid bacteria and their interference with human intestinal pathogen invasion. Gut Pathog..

[75] Heeney D.D., Gareau M.G., Marco M.L. (2018). Intestinal *Lactobacillus* in health and disease: A driver or just along for the ride?. Curr. Opin. Biotechnol..

[76] Kamada N., Chen G.Y., Inohara N., Núñez G. (2013). Control of pathogens and pathobionts by the gut microbiota. Nat. Immunol..

[77] Aljahdali N.H., Sanad Y.M., Han J., Foley S.L. (2020). Current knowledge and perspectives of potential impacts of *Salmonella enterica* on the profile of the gut microbiota. BMC Microbiol..

[78] Bridier A., Le Grandois P., Moreau M.H., Prénom C., Le Roux A., Feurer C., Soumet C. (2019). Impact of cleaning and disinfection procedures on microbial ecology and *Salmonella* antimicrobial resistance in a pig slaughterhouse. Sci. Rep..

[79] Ten Bruggencate S.J., Bovee-Oudenhoven I.M., Lettink-Wissink M.L., Van der Meer R. (2003). Dietary fructooligosaccharides dose-dependently increase translocation of *Salmonella* in rats. J. Nutr..

[80] Bovee-Oudenhoven I.M.J., Ten Bruggencate S.J.M., Lettink-Wissink M.L.G., Van der Meer R. (2003). Dietary fructooligosaccharides and lactulose inhibit intestinal colonization but stimulate translocation of *Salmonella* in rats. Gut.

[81] Mao B., Li D., Zhao J., Liu X., Gu Z., Chen Y.Q., Chen W. (2015). Metagenomic insights into the effects of fructooligosaccharides (FOS) on the composition of fecal microbiota in mice. J. Agric. Food Chem..

[82] Petersen A., Heegaard P.M., Pedersen A.L., Andersen J.B., Sørensen R.B., Frøkiær H., Licht T.R. (2009). Some putative prebiotics increase the severity of *Salmonella enterica* serovar Typhimurium infection in mice. BMC Microbiol..

[83] Tanno H., Fujii T., Hirano K., Maeno S., Tonozuka T., Sakamoto M., Endo A. (2021). Characterization of fructooligosaccharide metabolism and fructooligosaccharide-degrading enzymes in human commensal butyrate producers. Gut Microbes.

[84] Endo A., Tanno H., Kadowaki R., Fujii T., Tochio T. (2022). Extracellular fructooligosaccharide degradation in *Anaerostipes hadrus* for co-metabolism with non-fructooligosaccharide utilizers. Biochem. Biophys. Res. Commun..

[85] Maaroufi H., Levesque R.C. (2015). Glycoside hydrolase family 32 is present in *Bacillus subtilis* phages. Virol. J..

[86] Ahameethunisa A.R., Hopper W. (2010). Antibacterial activity of *Artemisia nilagirica* leaf extracts against clinical and phytopathogenic bacteria. BMC Complement. Altern. Med..

[87] Das Q., Lepp D., Yin X., Ross K., McCallum J.L., Warriner K., Marcone M.F., Diarra M.S. (2019). Transcriptional profiling of *Salmonella enterica* serovar Enteritidis exposed to ethanolic extract of organic cranberry pomace. PLoS ONE.

[88] Ma G., Chen Y. (2020). Polyphenol supplementation benefits human health via gut microbiota: A systematic review via meta-analysis. J. Funct. Foods.

